# A systematic review of obesity burden in Saudi Arabia: Prevalence and associated co-morbidities

**DOI:** 10.1016/j.jsps.2024.102192

**Published:** 2024-10-24

**Authors:** Hussain A. Al-Omar, Ali Alshehri, Saleh A. Alqahtani, Hana Alabdulkarim, Ali Alrumaih, Mahmoud S. Eldin

**Affiliations:** a Department of Clinical Pharmacy, College of Pharmacy, King Saud University, Riyadh 11451, Saudi Arabia; b Obesity Medicine Department, Obesity, Endocrine & Metabolism Centre, King Fahad Medical City, Riyadh, Saudi Arabia; c Liver Transplant Centre, King Faisal Specialist Hospital & Research Centre, Riyadh, Saudi Arabia; d Division of Gastroenterology and Hepatology, Johns Hopkins University, Baltimore, MD, USA; e Drug Policy and Economic Centre, Ministry of National Guards Health Affairs, Riyadh, Saudi Arabia; f Pharmaceutical Care Department, Medical Services Directorate, Ministry of Defence, Riyadh, Saudi Arabia; g Novo Nordisk, Riyadh, Saudi Arabia

**Keywords:** Obesity, Complications, Burden, Saudi Arabia

## Abstract

**Introduction:**

Saudi Arabia has experienced an increasing trend in obesity prevalence in the last three decades; obesity is a significant risk factor for non-communicable diseases, which may cause healthcare and economic burdens. In this systematic review, we aim to explore the obesity prevalence, obesity-related complications (ORCs), and the economic burden of obesity in Saudi Arabia.

**Methods:**

Literature searches for relevant local studies across Saudi Arabia spanning 2012 to 2022 were performed in PubMed and EMBASE, along with supplementary searches for relevant congress abstracts. Only studies that discussed obesity prevalence in Saudi Arabia in relation to any gender or age group, the prevalence of ORCs in Saudi Arabia for any gender or age group, and/or the economic burden of obesity and how it impacts the healthcare system in Saudi Arabia, and were published in the English language, were selected for inclusion. No age or gender restrictions were imposed.

**Results:**

The prevalence of obesity in Saudi Arabia ranged from 20% to 39% and up to 19.4% among adults and adolescents, respectively. The most reported ORCs were hypertension (67.6%), type 2 diabetes (60.7%), and hypercholesterolaemia (51.3%), and an association between obesity and ORCs was established, showing an increased risk with increasing body mass index. The economic burden of obesity across Saudi Arabia was estimated to be 6.4 billion US dollars (USD) for treatment and management.

**Conclusion:**

Obesity affects a substantial proportion of the Saudi general population and is a significant burden on individuals, as demonstrated by the prevalence of ORCs. Multifaceted, short- and long-term approaches involving interventions that operate at multiple levels and target both individuals and communities are urgently needed; there is a particular need for a national strategy and a specific, systems-based policy. Further research will help increase awareness of obesity and its management, which will be crucial for transforming the healthcare system under Vision 2030.

## 1 Introduction

Obesity is an abnormal or excessive fat accumulation that presents a health risk and is defined as a body mass index (BMI) of ≥ 30 kg/m^2^ (WHO, 2023). It is a complex disease with both non-modifiable and modifiable risk factors (Masood and Moorthy, 2023). Genetics is a significant non-modifiable factor (Keller et al., 2023), while epigenetics, physical inactivity, environmental factors, lifestyle choices, and medications which increase the likelihood of weight gain are considered among obesity top modifiable risk factors (Chakhtoura et al., 2023, Rohde et al., 2019). Additionally, several sociocultural factors were found to impact obesity burden including economic development, income, and cultural attitudes (Alsulami et al., 2023, Mosli et al., 2020). In Saudi Arabia, sedentary lifestyles, dietary changes, cultural factors, gender, and climate conditions contributed to the near-epidemic levels of obesity (Aldubikhi, 2023).

Worldwide, obesity prevalence has doubled in adults and quadrupled in adolescents since 1990 (WHO, 2023), with 2.7 billion adults expected to be overweight by 2025, 1 billion struggling with obesity, and 177 million having class III obesity (Mosli et al., 2020, World Obesity Federation, 2016). In children and adolescents, overweight or obesity prevalence has also increased, from 8 % to 20 % globally, with 37 million children below age 5 struggling with obesity (WHO, 2023). In Saudi Arabia, the World Health Survey (WHS–KSA), revealed a 20 % obesity prevalence among adults, with Al-Jawf, Bahah, and Najran having the highest prevalence (MOH, 2019). These findings were consistent with the findings from Saudi Health Interview Survey (Alluhidan et al., 2022).

Obesity is a significant risk factor for non-communicable diseases (NCDs) like cardiovascular disease (CVD), type 2 diabetes (T2D), infertility, and cancer (Haslam and James, 2005, Zain and Norman, 2008), putting a heavy burden on the healthcare system (Boutayeb and Boutayeb, 2005, Withrow and Alter, 2011). Moreover, obesity and its related complications (ORCs) have significant economic and societal impacts, including strains on healthcare systems and productivity loss (Tremmel et al., 2017). Economically, the impact of obesity was estimated to be 1.96 trillion USD with expectation to reach 4 trillion USD by 2035 (World Obesity Federation, 2023b). In Saudi Arabia, the economic impact of overweight and obesity was estimated to be 19.59 billion USD, equivalent to 572 USD per capita and 2.4 % of the gross domestic product (GDP) with expectations to increase to 150.46 billion USD, equivalent to 3,318 USD per capita and 5.6 % of GDP By 2060 (World Obesity Federation, 2023a).

The Arab region's rapid sociocultural and economic changes have led to increased obesity prevalence (Al-Rethaiaa et al., 2010). In Saudi Arabia, the cumulative expenditure on illness attributable to obesity is predicted to reach 127 billion USD by 2040 (Coker et al., 2022). To address this, Saudi Arabia has introduced a quality of life program focusing on physical activity and food habits, and introduced a new model of care that focus more on preventative approaches and services (Saudi Arabia Vision 2030). The Ministry of Health has implemented strategies to raise awareness, reduce sugary food intake, and provide dietary advices (Al Eid et al., 2017). Access to support and therapies for obesity and other chronic conditions has also improved. Early obesity management can reduce the risk of co-morbidities like CVD, T2D, OSA, and hypertension.

Understanding obesity prevalence, risk factors, and co-morbidities is crucial for tackling its burden on system, society and resources. Reducing sedentary behaviors and increasing physical activity can help in preventing obesity and improving health and well-being (Cameron et al., 2003, WHO, 2014). Early and effective obesity management can reduce ORCs risks (Cefalu et al., 2015, Montesi et al., 2016). Strategies to tackle obesity should focus on plans and policies to prevent and/or manage obesity and its complications and emphasizing on the impact of implementing early interventions both clinically and economically. Factors affecting obesity and co-morbidities are still debatable (Gadde et al., 2018); therefore, a robust scientific and clinical research is needed to address these debates. Geographical differences in data availability on obesity prevalence, such as in the Gulf region, necessitate regional studies (AlNohair, 2014). In Saudi Arabia, there is a scarcity in the local literature that discuss obesity and its complications from clinical and economical perspectives. Such an evidence could significantly assist stakeholders in calling for action and developing strategies and policies against obesity. Therefore, we conducted this systematic literature review (SLR) to explore obesity prevalence, ORCs, and the economic burden of obesity in Saudi Arabia.

## 2 Methods

### 2.1 Search strategy

The present SLR was conducted in accordance with the Preferred Reporting Items for Systematic Review and Meta-Analysis (PRISMA) in order to improve the quality and transparency of the studies (Page et al., 2021). A search for relevant literature was performed across PubMed, EMBASE, and the abstract database of the International Federation for the Surgery of Obesity and Other Metabolic Disorders (IFSO) 23rd World Congress in 2018, for the period between January 2012 and September 2021, with the last search update in December 2022. Only human-related studies published in the English language were included. No other restrictions, such as study design, gender, or age, were imposed. Searches of databases were carried out using a combination of medical subject heading terms and/or free text words using the following key search terms: obesity (terms in major indexing OR title) AND (geographical terms in title/abstract) AND (epidemiology terms OR cost/economic terms OR co-morbidity terms OR specific named conditions). For full search strategy and used keywords, see Appendix A. In addition, a manual search identified articles and congress abstracts relevant to the prevalence of obesity, the prevalence of OCRs, and the economic burden of obesity in Saudi Arabia that were not identified in the initial search.

More studies were searched in PubMed and EMBASE to obtain additional information about co-morbidities such as non-alcoholic fatty liver disease (NAFLD), polycystic ovary syndrome (PCOS), the link between obesity and Coronavirus disease (COVID-19), infertility, and mental health problems. The criteria for search strategy and population, intervention, comparison, outcomes, and study (PICOS) included children and adults in Saudi Arabia, and the prevalence of obesity and the prevalence of ORCs at any age and gender and in any study design (Supplementary Material).

### 2.2 Study selection

The inclusion criteria for studies were: studies related to obesity prevalence in Saudi Arabia of any gender or age group; studies related to ORCs in Saudi Arabia of any gender or age group; studies related to the economic burden of obesity in Saudi Arabia of any gender or age group; and studies published in the English language. All studies that did not meet these inclusion criteria were excluded.

### 2.3 Screening and data extraction

**Fig. 1:**
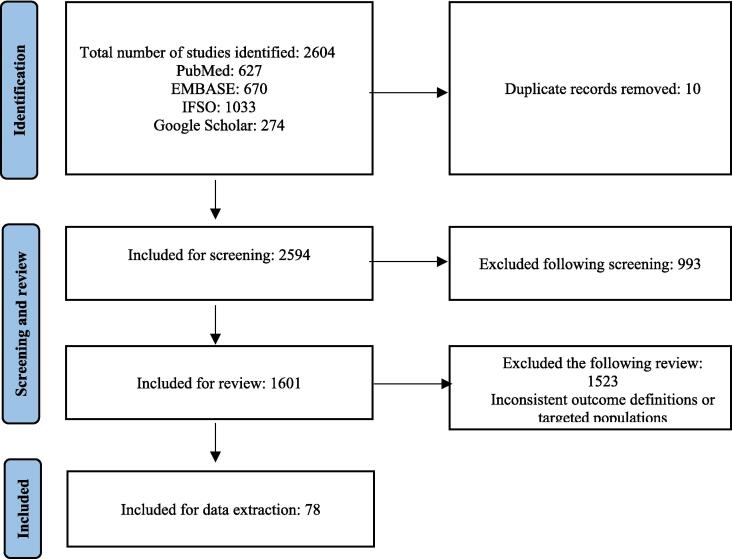
Preferred Reporting Items for Systematic Reviews and Meta-Analyses (PRISMA) flow diagram.

Studies obtained from the databases were screened for inclusion by three reviewers independently. All search results were exported into EndNote® software (Clarivate, Version 20, Philadelphia) and duplicates were removed. Then the remaining studies were screened for inclusion using a two-step approach: they were screened, first, by title and abstract for relevant information and, second, by retrieving and reviewing the study’s full text. After completing the screening, the reviewers met to compare their lists of included studies and resolve any disagreements. Disagreements were discussed among the reviewers and in each case a collective decision was made. The PRISMA flow chart (Fig. 1) shows the studies included following the screening based on the inclusion and exclusion criteria.

Data from the eligible studies and congress abstracts were manually extracted into a predefined data extraction form, using Microsoft Excel® software, including obesity prevalence by national estimate, sex, and age, prevalence of OCRs and healthcare resource utilization, and economic costs associated with treating obesity and/or its related complications. The extracted data from the different sources that matched our study aim and objectives were summarized in the form of tables. Subgroups of interest for the result synthesis included prevalence according to age, age group, age and sex, administrative region and for each ORCs.

### 2.4 Risk of bias assessment

Assessment of the risk of bias was performed as part of the data extraction using the Joanna Briggs Institute (JBI) criteria according to each study type. Any disagreement in quality assessment was resolved through consensus (Aromataris, 2020). For risk assessment risk assessment table for included studies see Appendix B.

## 3 Results

### 3.1 Systematic literature review

We identified 2,604 studies from the PubMed, EMBASE, and 2018 IFSO abstract database searches. Following the removal of 10 duplicate studies, 2,594 were included in the title and abstract screening. Following that screening, 994 studies were excluded due to the data not being relevant for Saudi Arabia, and 1,601 studies were further reviewed for relevance to the prevalence of obesity and ORCs and the economic burden of obesity on the healthcare system in Saudi Arabia. A total of 1,523 studies were excluded after reviewing due to inconsistent outcome definitions and targeted populations. Finally, 78 studies were included for data extraction (Fig. 1). A list of excluded records can be found in Appendix C. Of the 78 studies remaining for full analysis, 59 were cross-sectional, one was experimental, one was a survey, four were prospective and 13 were retrospective. The sample size for these studies ranged from 80 to 615,768 individuals. In total, 31 studies discussed obesity prevalence, 45 studies discussed the prevalence of ORCs in people with obesity (PwO) and three discussed the economic burden of obesity on the health system.

### 3.2 Obesity prevalence in Saudi Arabia stratified by age

Sixteen studies reported obesity prevalence in adults and 15 reported obesity prevalence in children and adolescents (aged ≤ 19 years; Table 1). The prevalence of obesity in adults ranged from 24.4 % to 57 % (Ahmed et al., 2017, Ahmed et al., 2014, Al-Daghri et al., 2015, Al-Raddadi et al., 2019, Al-Sumaih et al., 2020, Alammar et al., 2020, Alghamdi et al., 2017, Alghnam et al., 2021, Alsaghah et al., 2019, AlShahrani, 2021, Althumiri et al., 2021; Alzeidan et al.; Azzeh et al., 2017, Habib, 2013, Memish et al., 2014, Mosli et al., 2020). The highest obesity prevalence estimate of 57 % was reported in a study conducted in the Bisha region (AlShahrani, 2021), while the lowest obesity prevalence estimate of 20.3 % was reported in Hail city in a cross-sectional survey (Ahmed et al., 2014). We identified the obesity prevalence in children and adolescents across 15 studies as ranging from 13.4 % to 86.6 % (Al-Agha et al., 2016b, Al-Daghri et al., 2015, Al-Hazzaa et al., 2014, Al-Hussaini et al., 2019, Al-Hussein et al., 2014, Al-Kutbe et al., 2017, Al-Nakeeb et al., 2012, AlAlwan et al., 2013, AlBuhairan et al., 2015, Bandy et al., 2019, Elkhodary and Farsi, 2017, Farsi and Elkhodary, 2017, Farsi et al., 2016, Nasim et al., 2019, Wagdy et al., 2021) (Table 1). A study of 541 children attending ambulatory clinics in Jeddah reported the highest obesity prevalence among children and adolescents (86.6 %) (Al-Agha et al., 2016b). The lowest obesity prevalence estimate (13.4 %) was reported in a cross-sectional study in Riyadh among 1,212 children and adolescents (AlAlwan et al., 2013).

**Table 1:** Prevalence of overall obesity in adults and children/adolescents.

**Study**	**Region**	**Study design**	**Sample size**	**Age** **(mean ± SD or range) (years)**	**Prevalence of obesity reported (%)**
***Adults***					
(Mosli et al., 2020)	Jeddah	Cross-sectional	3925	NR	25.8
(Ahmed et al., 2017)	Riyadh	Cross-sectional	2095	NR	39.1
(Ahmed et al., 2014)	Hail	Survey	5000	43.5 ± 18.7	20.3
(Al-Daghri et al., 2015)	Riyadh	Cross-sectional	830	36.8	38.5
(Alammar et al., 2020)	Riyadh	Cross-sectional	942	55.7	41
(Alghamdi et al., 2017)	Riyadh	Cross-sectional	160	34.4	24.4
(Alghnam et al., 2021)	National	Cross-sectional	615,768	≥17	38.96
(Al-Raddadi et al., 2019)	Jeddah	Cross-sectional	1134	NR	35.2
(Alsaghah et al., 2019)	National	Cross-sectional	1065	≥21–<60	29.5
(AlShahrani, 2021)	Bisha	Cross-sectional	612	≥20–<80	57
(Al-Sumaih et al., 2020)	National	Cross-sectional	2084	39.2	35.2
(Alzeidan et al., 2020)	Riyadh	Cross-sectional	3063	38.58 ± 14.34	36.8
(Althumiri et al., 2021)	National	Cross-sectional	4709	36.4 ± 13.5	21.7
(Azzeh et al., 2017)	Makkah	Cross-sectional	2548	29.1 ± 8.5	25.5
(Habib, 2013)	Riyadh	Cross-sectional	428	36.90 ± 15.22	28
(Memish et al., 2014)	National	Cross-sectional	10,735	≥15	32.2
***Children and adolescents***					
(Al-Daghri et al., 2015)	Riyadh	Cross-sectional	2225	15.1 ± 0.06	15.1
(Al-Agha et al., 2016b)	Jeddah	Cross-sectional	541	10.1	86.6
(Alwan İbrahim et al., 2013)	Riyadh	Cross-sectional	1212	6–16	13.4
(AlBuhairan et al., 2015)	National	Cross-sectional	12,575	9–18	15.9
(Al-Hazzaa et al., 2014)	National	Cross-sectional	2908	16.7 ± 1.1	19
(Al-Hussaini et al., 2019)	Riyadh	Cross-sectional	7930	11.1 ± 2.64	18.2
(Al-Hussein et al., 2014)	Riyadh	Cross-sectional	2149	NR	14.8
(Al-Kutbe et al., 2017)	Makkah	Cross-sectional	266	8–11	17.3
(Al-Nakeeb et al., 2012)	National	Cross-sectional	1118	15–17	18.3
(Bandy et al., 2019)	Al-Jouf	Cross-sectional	400	15–17	33.5
(Elkhodary and Farsi, 2017)	Jeddah	Cross-sectional	1523	7–19	20.8
(Farsi and Elkhodary, 2017)	Jeddah	Cross-sectional	801	16.5 ± 0.9	24
(Farsi et al., 2016)	Jeddah	Cross-sectional	915	9.0 ± 8.9	18
(Nasim et al., 2019)	Riyadh	Cross-sectional	481	<10	18.2
(Wagdy et al., 2021)	Jeddah	Cross-sectional	299	5–13	13.7

Obesity prevalence (%), an average between men and women as reported in previous studies, NR, not reported; SD, standard deviation

### 3.3 Obesity prevalence in Saudi Arabia stratified by age group

Few studies have examined the prevalence of obesity across multiple age groups in Saudi Arabia (Table 2). Four studies stratified obesity based on age group but used different age group thresholds. Overall, obesity prevalence increased with age, ranging from 14.84 %–39.5 % in individuals aged 18 − 45 years, 29.8 %–50.0 % in individuals aged 40 − 49 years, and 27.6 %−69.2 % in individuals aged ≥ 50 years (Al-Kadi et al., 2018, Aldiab et al., 2018, AlShahrani, 2021, Althumiri et al., 2021).

**Table 2:** Prevalence of obesity stratified by age group.

**Study**	**Region**	**Type of study design**	**Sample size**	**Age(mean ± SD or range) (years)**	**Prevalence of obesity by age (%)**
(AlShahrani, 2021)	Bisha	Cross-sectional	612	<20–80	<20, 0.65; 21–40, 8.3; 41–60, 27.4; 61–80, 19.6; >80, 1.8
(Althumiri et al., 2021)	National	Cross-sectional	4709	36.4 ± 13.5	18–19 years, 14.1; 20–29 years, 14.8; 30–39 years, 18.1; 40–49 years, 29.8; 50–59 years, 32.8; ≥60 years, 27.6
(Al-Kadi et al., 2018)	Al-Qassim	Cross-sectional	646	30.13 ± 12.15	<20 years, 28.7; 20–39 years, 33.4; 40–60 years, 61.9
(Aldiab et al., 2018)	Al-Kharj	Cross-sectional	1019	NR	18–29 years, 14.8; 30–39 years, 15.5; 40–49 years, 13.7; 50–67 years, 26.3

NR, not reported; SD, standard deviation

### 3.4 Obesity prevalence in Saudi Arabia stratified by age and sex

Fourteen studies reported obesity prevalence by sex among adults; obesity prevalence ranged from 18.1 % to 38.3 % in men and from 11.2 % to 53 % in women (Al-Daghri et al., 2015, Al-Kadi et al., 2018, Al-Qahtani, 2019, Al-Raddadi et al., 2019, Al-Sumaih et al., 2020, Alammar et al., 2020, AlShahrani, 2021, Althumiri et al., 2021, Alwasaidi et al., 2017, Alzeidan et al., 2020, Azzeh et al., 2017, Memish et al., 2014, Mosli et al., 2020) (Table 3). Of the 14 studies, nine reported a higher prevalence of obesity in women than men. Among men, the highest prevalence was reported in a cross-sectional study (n = 1,681) in the southwest of Saudi Arabia, with an estimated prevalence of 38.3 % (Al-Qahtani, 2019), while in women the highest prevalence (53 %) was reported in 2020 (n = 942) in Riyadh city (Alammar et al., 2020).

**Table 3:** Obesity prevalence in Saudi Arabia stratified by age and sex.

**Study**	**Region**	**Type of study design**	**Sample size**	**Age** **(mean ± SD or range) (years)**	**Prevalence in men (%)**	**Prevalence in women (%)**
***Adult men and women***						
(Mosli et al., 2020)	Jeddah	Cross-sectional	3925	NR	26.68	25.16
(Al-Daghri et al., 2015)	Riyadh	Cross-sectional	830	18–50	33.6	43.4
(Alammar et al., 2020)	Riyadh	Cross-sectional	942	56	29.2	53
(Al-Raddadi et al., 2019)	Jeddah	Cross-sectional	1134	NR	34.8	35.6
(AlShahrani, 2021)	Bisha	Cross-sectional	612	20–80	23.5	34.1
(Al-Sumaih et al., 2020)	National	Cross-sectional	2084	39.2	31.59	39.5
(Alzeidan et al., 2020)	Riyadh	Cross-sectional	3063	38.58 ± 14.34	33.1	39
(Althumiri et al., 2021)	National	Cross-sectional	4709	36.4 ± 13.5	17.9	25.5
(Azzeh et al., 2017)	Makkah	Cross-sectional	2548	18 − 60	29.3	21.7
(Memish et al., 2014)	National	Cross-sectional	10,735	≥15	27.8	38.8
(Al-Kadi et al., 2018)	Al-Qassim	Cross-sectional	646	30.13 ± 12.15	34.2	41.7
(Al-Qahtani, 2019)	South-west	Cross-sectional	1681	74.1 ± 15.81	38.3	27.6
(Alwasaidi et al., 2017)	Al-Madinah	Cross-sectional	1171	15–55	35.7	20.3
(Aldiab et al., 2018)	Al-Kharj	Cross-sectional	1019	NR	18.1	11.2
***Children and adolescent boys and girls***						
(Al-Daghri et al., 2015)	Riyadh	Cross-sectional	2225	13–17	17.8	12.4
(Alwan İbrahim et al., 2013)	Riyadh	Cross-sectional	1212	6–16	17.4	9.3
(AlBuhairan et al., 2015)	National	Cross-sectional	12,575	9–18	20.2	11
(Al-Hazzaa et al., 2014)	National	Cross-sectional	2908	16.7 ± 1.1	24.1	14
(Al-Hussaini et al., 2019)	Riyadh	Cross-sectional	7930	11.1 ± 2.64	18.4	18
(Al-Nakeeb et al., 2012)	National	Cross-sectional	1118	16.6	19.5	17.1
(Farsi et al., 2016)	Jeddah	Cross-sectional	915	9	20	16
(Alshammari et al., 2017)	Hail	Cross-sectional	1420	2–18	10	12.55
(Al-Enazy et al., 2014)	Tabuk	Cross-sectional	350	6–13	17.4	20.9
(Al-Mohaimeed et al., 2015)	Al-Qassim	Cross-sectional	874	8.4 ± 1.1	17.3	34.3
(Al-Mohaimeed, 2016)	Al-Qassim	Cross-sectional	601	8.4 ± 1.3	14.1	7.2
(Ashi et al., 2019)	Jeddah	Cross-sectional	669	13–15	43.9	20.7
(Shaikh et al., 2016)	Al-Qassim	Cross-sectional	242	12–14	11.8	8.8

^a^Class I obesity, 30–34.9 kg/m^2^; ^b^Class II/III obesity, ≥35 kg/m^2^.

NR, not reported; SD, standard deviation

In children and adolescents, obesity prevalence was reported in 13 studies, ranging from 10 % to 43.9 % in boys and from 8.4 % to 34.3 % in girls (Al-Daghri et al., 2015, Al-Enazy et al., 2014, Al-Hazzaa et al., 2014, Al-Hussaini et al., 2019, Al-Mohaimeed et al., 2015, Al-Mohaimeed, 2016, Al-Nakeeb et al., 2012, AlAlwan et al., 2013, AlBuhairan et al., 2015, Alshammari et al., 2017, Ashi et al., 2019, Farsi et al., 2016, Shaikh et al., 2016). Obesity prevalence was more significant in boys than in girls in 10 studies, while three studies reported a higher prevalence of obesity in girls than in boys. A cross-sectional study of 669 children in Jeddah found that boys had a higher prevalence (43.9 % vs 20.7 %) than girls (Ashi et al., 2019). In comparison, the highest prevalence (34.4 % vs 17.3 %) among girls was reported in a cross-sectional study among 874 adolescents in the Al-Qassim region (Al-Mohaimeed et al., 2015).

### 3.5 Obesity prevalence in Saudi Arabia stratified by administrative region

We assessed the prevalence of obesity according to province. In one study, the highest levels of prevalence of obesity across the 13 administrative regions of Saudi Arabia were in Riyadh and the Eastern region (26.9 % and 29.4 %, respectively), while the lowest prevalence levels were in Baha and Asir provinces (14.3 % and 18.0 %, respectively). The remaining nine regions reported prevalence estimates between 18.0 % and 26.9 % (Althumiri et al., 2021).

### 3.6 Orcs in Saudi Arabia

We identified several studies through the SLR that assessed the prevalence of ORCs, including T2D, CVD, NAFLD, OSA, infertility, PCOS, and osteoarthritis (Table 4).

**Table 4:** Prevalence of ORCs among PwO.

**Study**	**Region**	**Type of study design**	**Sample size**	**Age (mean ± SD or range) (years)**	**Prevalence of obesity reported (%)**	**Prevalence of ORCs reported among PwO (%)**
***Impaired glucose regulation: Diabetes***						
(Alghnam et al., 2021)	National	Cross-sectional	615,768	≥17	38.96	26.8
(Althumiri et al., 2021)	National	Cross-sectional	4709	NR	21.7 (n = 1023)	10.2
(Memish et al., 2014)	National	Cross-sectional	10,735	≥15	32.2 (n = 3455)	18
(Aleidi et al., 2021)	Riyadh	Experimental	101	34.6–49.4	51.4 (n = 52)	50
(Aljabri et al., 2018)	Jeddah	Cross-sectional	2452	45.7	100	37.9
(Almojarthe et al., 2020)	Abha	Cross-sectional	198	31.6	100	15.7
(Alqahtani et al., 2012)	Riyadh	Retrospective	108	13.9 ± 4.3	100	20.4
(Al-Zahrani et al., 2019)	Al-Kharj	Cross-sectional	638	23.3	22.57 (n = 144)	11.8
(Assakran et al., 2020)	Al-Qassim	Cross-sectional	98	35	100	10.2
(Mobeirek et al., 2014)	National	Prospective	3469 ACS	57	31.1 (n = 1081)	60.7
***Impaired glucose regulation: Prediabetes***						
(Alqahtani et al., 2012)	Riyadh	Retrospective	108	13.9 ± 4.3	100	13.0
(Al-Zahrani et al., 2019)	Al-Kharj	Cross-sectional	638	23.3	22.57 (n = 144)	31.2
(Alhazmi et al., 2017)	Al-Turaif	Cross-sectional	402	23.27	22.2 (n = 89)	12.3
***Hypertension***						
(Alghnam et al., 2021)	National	Cross-sectional	615,768	≥17	38.96 (n = 239,913)	23.8 (n = 57,165)
(Alsaghah et al., 2019)	National	Cross-sectional	1065	<21-≥60	29.5 (n = 315)	20.9
(Althumiri et al., 2021)	National	Cross-sectional	4709	36.4 ± 13.5	21.7 (n = 1022)	22.7 (n = 232)
(Memish et al., 2014)	National	Cross-sectional	10,735	≥15	32 (n = 3342)	29.4
(Wagdy et al., 2021)	Jeddah	Cross-sectional	299	5–17	13.7 (n = 41)	36.5
(Aljabri et al., 2018)	Jeddah	Cross-sectional	2452	45.7	100	21.9
(Almojarthe et al., 2020)	Abha	Cross-sectional	198	31.6	100	12.1
(Alqahtani et al., 2012)	Riyadh	Retrospective	108	13.9 ± 4.3	100	36.1 (n = 39)
(Mobeirek et al., 2014)	National	Prospective	3469	57	22 (n = 762)	67.6
***Cardiovascular diseases/dyslipidemia/hyperlipidemia***						
(Althumiri et al., 2021)	National	Cross-sectional	4709	36.4 ± 13.5	21.7 (n = 1022)	17.6 (n = 180)
(Memish et al., 2014)	National	Cross-sectional	10,735	15–65	31.1 (n = 3364)	12
(Aldiab et al., 2018)	Al-Kharj	Cross-sectional	1019	18–67	27.6 (n = 281)	11
(Almojarthe et al., 2020)	Abha	Cross-sectional	198	NR	100	8.6
(Alqahtani et al., 2012)	Riyadh	Retrospective	108	13.9 ± 4.3	100	48.1 (n = 52)
(Mobeirek et al., 2014)	National	Prospective	3469	57	22 (n = 762)	51.3
***Non-alcoholic fatty liver disease***						
(Althumiri et al., 2021)	National	Cross-sectional	4709	36.4 ± 13.5	21.7 (n = 1022)	1.1 (n = 11)
(Alsabaani et al., 2018)	Abha	Cross-sectional	245	57.1	40.4 (n = 99)	78.8
(Faisal et al., 2016)	Makkah	Cross-sectional	300	≥18	53.6 (n = 161)	85.1
***Sleep disorders***						
(Althumiri et al., 2021)	National	Cross-sectional	4709	36.4 ± 13.5	21.7 (n = 1022)	2.3 (n = 24)
(Alqahtani et al., 2012)	Riyadh	Retrospective	108	13.9 ± 4.3	100	33.3 (n = 36)
(Alshehri et al., 2019)	Jeddah	Cross-sectional	803	45.9 ± 15.9	70.4 (n = 564)	82.8
***Infertility***						
(Rafique and Nuzhat, 2016)	Riyadh	Retrospective	826 females	≤42	36.3	100
(Rafique et al., 2021)	Riyadh	Retrospective	196 females	17–40	48	100
(Alshahrani et al., 2016)	Al-Kharj	Prospective	439 males	≥18	45.5	100
***Osteoarthritis/Knee injuries***						
(Alanazi et al., 2022)	Hail	Cross-sectional	172	57	100	76
(AlKuwaity et al., 2018)	Arar	Cross-sectional	229	70.4	43.7 (n = 104)	25 (n = 26)
(Almaawi et al., 2020)	Riyadh	Cross-sectional	482	21.5	23.2	16.9 (n = 19)

n, number; NR, not reported; ORC, obesity-related complication; PwO, people with obesity; SD, standard deviation

#### 3.6.1 T2D

In total, 11 studies examined the prevalence of T2D, and the estimated prevalence of T2D among PwO patients ranged from 4.5 % to 60.7 % (Al-Zahrani et al., 2019, Aleidi et al., 2021, Alghnam et al., 2021, Alhazmi et al., 2017, Aljabri et al., 2018, Almojarthe et al., 2020, Alqahtani et al., 2012, Althumiri et al., 2021, Assakran et al., 2020, Memish et al., 2014, Mobeirek et al., 2014). The highest prevalence of T2D (60.7 %) among PwO was reported in a prospective national study (n = 3,469) of patients with acute coronary syndrome (ACS). The lowest prevalence (4.5 %) was reported in a cross-sectional study of 402 subjects in Turaif city (Alhazmi et al., 2017). The number of people with pre-diabetes was examined in three studies, and the results ranged from 12.3 % to 31.2 % (Al-Zahrani et al., 2019, Alhazmi et al., 2017, Alqahtani et al., 2012) (Table 4).

##### 3.6.1.1 CVD

Nine studies examined the prevalence of hypertension in PwO patients, and the outcomes ranged from 12.1 % to 67.6 % (Alghnam et al., 2021, Aljabri et al., 2018, Almojarthe et al., 2020, Alqahtani et al., 2012, Alsaghah et al., 2019, Althumiri et al., 2021, Memish et al., 2014, Mobeirek et al., 2014, Wagdy et al., 2021). The highest prevalence was 67.6 % (Mobeirek et al., 2014), and the lowest prevalence was 12.2 %, as reported in a cross-sectional study (n = 198) in Abha city (Almojarthe et al., 2020) (Table 4). In addition, six studies highlighted the interlinkage between obesity and hypercholesterolaemia; the prevalence ranged from 8.6 % to 51.3 % among PwO (Almojarthe et al., 2020, Alqahtani et al., 2012, Alsabaani et al., 2018, Althumiri et al., 2021, Memish et al., 2014, Mobeirek et al., 2014), with the highest (51.3 %) reported in a prospective national study of 3,469 patients with ACS (Mobeirek et al., 2014), and the lowest (8.6 %) reported in a cross-sectional study in Abha city (n = 1,019) (Almojarthe et al., 2020) (Table 4).

##### 3.6.1.2 NAFLD

The prevalence of NAFLD or non-alcoholic steatohepatitis with either BMI or other ORCs was addressed in three studies, with a wide prevalence ranging from 1.1 % to 85.1 % among PwO (Alsabaani et al., 2018, Althumiri et al., 2021, Faisal et al., 2016). In a study in Makkah city (n = 300), the prevalence of NAFLD among PwO was 85.1 % (Faisal et al., 2016), while another national cross-sectional study (n = 4,707) showed that only 1.1 % of PwO reported liver diseases (Althumiri et al., 2021) (Table 4).

##### 3.6.1.3 OSA

The prevalence of OSA among PwO in Saudi Arabia’s populations was estimated in three studies and ranged from 2.3 % to 82.8 % (Alqahtani et al., 2012, Alshehri et al., 2019, Althumiri et al., 2021). The highest prevalence of OSA (82.8 %) among PwO patients was reported in a cross-sectional study of 803 patients in Jeddah city (Alshehri et al., 2019). However, OSA has been reported in only 2.3 % of PwO in a national study (n = 4,707) (Althumiri et al., 2021) (Table 4).

##### 3.6.1.4 Infertility

Three studies investigated the link between infertility and obesity in Saudi Arabia; two studies examined women’s infertility retrospectively (Rafique et al., 2021, Rafique and Nuzhat, 2016), while one prospective study examined infertility issues among men (Alshahrani et al., 2016) (Table 4). The men’s infertility study examined different aspects of sperm quality with BMI; 45.5 % of PwO were reported to have infertility (Alshahrani et al., 2016). In the women’s studies, infertility among PwO ranged from 36.3 % to 48 % (Rafique et al., 2021, Rafique and Nuzhat, 2016).

##### 3.6.1.5 Osteoarthritis/knee injuries

Three studies investigated increased BMI and its associated risk in terms of the prevalence of osteoarthritis or knee injuries; the prevalence of osteoarthritis or knee injuries ranged from 16.9 % to 76 % in PwO (Alanazi et al., 2022, AlKuwaity et al., 2018, Almaawi et al., 2020). A cross-sectional study (n = 172) reported the highest prevalence of osteoarthritis (76 %) in Hail city (Alanazi et al., 2022). In contrast, the lowest prevalence (16.9 %) of knee injuries was reported among PwO in Riyadh city in a cross-sectional study (Almaawi et al., 2020) (Table 4).

### 3.7 Association of several disease areas and obesity in Saudi Arabia

Four studies that examined the association between breast cancer and obesity in Saudi Arabia were identified in our report; across these studies, the prevalence of obesity in association with the different grades and stages of breast cancer ranged from 46.6 % to 81.3 % (Al-Saleh et al., 2019, Alshamsan et al., 2022, Elkum et al., 2014, Rasmy and Sorour, 2020). The highest obesity prevalence among patients with breast cancer was presented in a national cross-sectional study (n = 246), with an average obesity prevalence of 81.3 % from the whole sample of patients (Al-Saleh et al., 2019) (Table 5).

**Table 5:** Association of several disease areas and obesity.

**Study**	**Region**	**Type of study design**	**Sample size**	**Age (mean ± SD or range) (years)**	**Prevalence of obesity (%)**
***Breast cancer***					
(Al-Saleh et al., 2019)	National	Retrospective	246	24–68	81.3
(Alshamsan et al., 2022)	Riyadh	Retrospective	2212	45.7	53.4
(Elkum et al., 2014)	Riyadh	Case-control	538	43.6 ± 8.3	46.40
(Rasmy and Sorour, 2020)	Dammam	Retrospective	80	50.8 ± 7.8	55
***COVID-19***					
(Al-Omari et al., 2020)	Riyadh	Cross-sectional	401	1.5–76	11.5
(Abohamr et al., 2020)	Riyadh	Case-series	768	46.36 ± 13.7	18.6
(Alguwaihes et al., 2020)	Riyadh	Retrospective	439	55	42.2
(Alharbi et al., 2022)	Southern	Retrospective	809	60 ± 17.7	43
(AlJabr et al., 2021)	Dammam	Retrospective	639	18-≥70	47.1
(AlKhafaji et al., 2022)	Dammam	Retrospective	502	<20-≥80	48.6
(Alqahtani et al., 2022)	Riyadh	Prospective	598	57	43
(Alqahtani et al., 2022)	Riyadh	Retrospective	951	0-≥61	29.5
(Melebari et al., 2021)	Makkah	Retrospective	369	≥18	45.8
***Polycystic ovary syndrome***					
(Al-Ruthia et al., 2017)	Riyadh	Retrospective	109	27	59.63
(Alsibyani et al., 2017)	Jeddah	Cross-sectional	183	18–45	67.2
(Altamimi et al., 2020)	Riyadh	Prospective	99	20–51	52.5
(Ibrahim et al., 2017)	Riyadh	Case-control	122	14–45	26.2

ORC, obesity-related complication; PwO, people with obesity; SD, standard deviation

Conversely, we assessed the COVID-19 pandemic in relation to BMI and obesity across several studies in Saudi Arabia. In our review, we identified nine studies that highlighted the impact and prevalence of obesity among patients with COVID-19. The overall prevalence of obesity among patients with COVID-19 ranged from 11.5 % to 48.6 % (Abohamr et al., 2020, Al-Numair et al., 2022, Alguwaihes et al., 2020, Alharbi et al., 2022; AlJabr et al.; AlKhafaji et al., 2022, Alqahtani et al., 2022, Melebari et al., 2021). The highest obesity prevalence (48.6 %) was reported in a retrospective study in Dammam city (n = 502) (AlKhafaji et al., 2022) (Table 5).

We also found that PCOS was identified as an ORC, it was investigated in four studies. The prevalence of PCOS among PwO patients ranged from 26.2 % to 67.2 % (Al-Ruthia et al., 2017, Alsibyani et al., 2017, Altamimi et al., 2020, Ibrahim et al., 2017). The highest prevalence (67.2 %) was reported among 183 patients in a cross-sectional study in Jeddah (Alsibyani et al., 2017). In contrast, 26.2 % of PwO in a case-controlled study of 122 participants were diagnosed with PCOS (Ibrahim et al., 2017) (Table 5).

### 3.8 Consequences of obesity on mental health

We explored five studies among children, adolescents, and adults that highlighted the consequences of obesity for mental health. The mental health areas included depression, anxiety, stress, and sadness (Al-Agha et al., 2016a, AlBuhairan et al., 2015, Almarhoon et al., 2021, AlQahtani et al., 2015, Alsaleem, 2021). Stress in the daily lives of PwO was presented in three studies, it ranged between 46 % and 57.7 % (Al-Agha et al., 2016a, AlQahtani et al., 2015, Alsaleem, 2021) among PwO (Table 6). Anxiety and depression were presented in two studies (AlQahtani et al., 2015, Alsaleem, 2021), and three out of the four studies on PwO were concerned with anxiety, while depression was reported among PwO with a range of 41.7 % to 65.7 %, across three studies (Almarhoon et al., 2021, AlQahtani et al., 2015, Alsaleem, 2021) (Table 6).

**Table 6:** Effects of obesity on mental health among PwO and children and adolescents with obesity.

**Study**	**Region**	**Type of study design**	**Sample size**	**Age (mean ± SD or range) years**	**Prevalence of obesity (%)**	**Prevalence of Psychiatric disorder (%)**
***Mental health***						
(AlBuhairan et al., 2015)	National	Cross-sectional	12,575	9–18	15.6 (n = 1962)	Feeling sad/hopeless: 14.3 Feeling worried: 6.7
(Al-Agha et al., 2016a)	Jeddah	Cross-sectional	281	Male: 10.6 ± 3.9; Female: 10.5 ± 3.8	100 (n = 281)	Walking problem: 50.6 Running: 55.8 Severe stress: 46 Maintain attention: 37.4 Follow-up duties: 31.3
(AlQahtani et al., 2015)	Abha	Cross-sectional	389	21.2	18.3 (n = 77)	Depression: 59.2 Anxiety: 74.6 Stress: 57.7
(Almarhoon et al., 2021)	Eastern	Cross-sectional	711	18–65	26.4 (n = 187)	Depression: 41.7
(Alsaleem, 2021)	Abha	Cross-sectional	398	12–18	38.4 (n = 153)	Depression: 65.7 Anxiety: 73.2 Stress: 44.4

n, number; PwO, people with obesity; ALwO, adolescents with obesity; SD, standard deviation

### 3.9 Economic burden of obesity in Saudi Arabia

Few studies have assessed the economic burden of obesity and how healthcare resources are utilized to manage obesity and its complications for individuals, healthcare systems, and institutions. A study from a hospital in Al-Qassim discovered that the average cost of laparoscopic sleeve gastrectomy with or without routine post-sleeve gastrografin was £5,193.15 (± £1,054.77) or £4,222.27 (± £857.58), respectively, and the average length of hospital stay was three days in both groups (Assakran et al., 2020).

A supplementary search identified a report from the World Obesity Federation that estimated that 13.6 % of the total healthcare expenditure, translating to 147.8 billion USD, was due to high BMI in the Eastern Mediterranean region. Similarly, Saudi Arabia was estimated to have healthcare costs of 6.4 billion USD attributed to obesity in 2016, with a predicted gross domestic product loss of 4.4 % owing to obesity, which is the fifth highest worldwide (World Obesity Federation, 2020a).

## 4 Discussion

The collation of available data published since 2012 in our study provides important insights into the prevalence of obesity and ORCs, as well as the age, regional, and sex differences in obesity prevalence, and the economic burden of obesity on the healthcare system in Saudi Arabia. The rising prevalence of obesity is a growing concern in Saudi Arabia, with rates of obesity having increased significantly over the past three decades (Alfaifi et al., 2020, Rakic et al., 2022). This trend highlights the need for continued efforts to address the issue of obesity in the country and promote healthy lifestyles among the population; however, there is currently a gap in the knowledge and misperceptions about obesity, and a lack of interest in the prevalence and impact of obesity and its related complications among healthcare providers and PwO in Saudi Arabia.

The prevalence of obesity in Saudi Arabia reported in the identified studies is high compared to the global estimates of obesity (WHO, 2023), although the population of PwO in Saudi Arabia has increased only relatively recently. One reason is that more people are interested in the Western way of life and diet, and with that comes a higher caloric intake from eating fast food (Alfaifi et al., 2020). In recent years, Saudi Arabia has experienced a major shift in dietary habits, moving away from its traditional diet towards a Western diet that is high in animal fats and refined sugars. This change in dietary patterns has contributed to the increasing obesity prevalence in the country. As more and more people adopt unhealthy eating habits, the rates of overweight and obesity are continuing to rise, highlighting the need for interventions to promote healthier dietary choices among the population (Alfawaz et al., 2020, Allam et al., 2012).

The SLR results also indicate that the prevalence of obesity in Saudi Arabia increases with age. Being aware of this should prompt faster actions to prevent and treat obesity earlier, in order to decrease the clinical and economic burdens of obesity. In addition, most identified adult studies found that women have a higher obesity prevalence than men, which should help shape community readiness campaigns that target each population group to better predict outcomes. Notably, the reported prevalence of obesity among Saudi Arabian women is higher than that among women globally (WHO, 2023).

We identified only one study that examined obesity prevalence by region in Saudi Arabia, and the results suggest that there are regional differences in obesity prevalence, with the highest prevalence observed in Riyadh and the Eastern region. This may be due to the particular climate and terrain found in cities or to provincial differences in food habits, lifestyles, and behaviors, or a combination of both; it requires further investigation (Althumiri et al., 2021). These results could help us learn more about the causes of obesity, especially regarding the social and economic conditions of cities in Saudi Arabia, and thus to tailor the most effective targeted interventions to tackle obesity. In some rural areas of Saudi Arabia, the prevalence of obesity has been reported to be only 4 % (Al-Ghamdi et al., 2018). Obesity has worsened in Saudi Arabia, mainly among women, and is now one of the leading causes of heart disease and T2D (Mobeirek et al., 2014). It has also been linked to numerous NCDs (Althumiri et al., 2021). It is predicted that overweight or obese Saudi children will carry the burden of excessive weight and the associated disabilities into adulthood (Aljassim and Jradi, 2021).

Several studies found through this SLR and other reported that OCRs are common in Saudi Arabia. The OCR with the highest prevalence in PwO is OSA, followed by hypertension, hypercholesterolaemia, and T2D. Several studies have also described a link between obesity, being overweight, or having a high BMI and complications such as infertility, PCOS, risk of developing breast cancer, and aspects of poor mental health, including feelings of worry, social problems, attention problems, psychological distress, and depression. Notably, the studies identified in this review, which examined the relationship between obesity and COVID-19, did not report any evidence to suggest an increased predisposition to severe COVID-19 in PwO compared with those without obesity. However, recent studies investigating the link between obesity and a number of ORCs and their markers, including high serum triglycerides, total cholesterol, low density–lipoprotein cholesterol, blood pressure, ferritin, and low relative lymphocyte count, found them to be associated with increased risk of infection with a more severe form of COVID-19, as well as increased risk for mortality and intubation compared to those with normal BMI (Mehanna et al., 2021, Nakeshbandi et al., 2020).

Other findings in our SLR were linked to the estimated odds ratio (OR) for the increased incidence of ORCs among PwO. The estimated OR for the development of T2D in PwO was 1.52, while the estimated OR for the development of hypertension was 1.61, which was consistent with the estimated ORs for developing hypercholesterolaemia in PwO, ranging from 1.69 to 2.60. We also assessed OSA towards its estimated OR with an estimated outcome of 1.82 (Althumiri et al., 2021).

There is a lack of evidence in the SLR regarding the economic burden and healthcare resource utilization associated with obesity and its related complications in Saudi Arabia. These outcomes should help key stakeholders, healthcare societies, and physicians call for action towards better healthcare for PwO in Saudi Arabia, improving the quality of citizen’s lives and contributing to Vision 2030 (Saudi Arabia Vision 2030, 2021). Subsequently, a targeted supplementary search was performed, identifying one report from the World Obesity Federation that describes increased healthcare expenditure and lost GDP associated with obesity in Saudi Arabia (World Obesity Federation, 2020b). This highlights a critical evidence gap that warrants further research. Overall, there is a clear economic case for preventing and treating obesity as early as possible. Using economic evaluation and modelling techniques as part of research can support resource allocation at the local level, enabling Saudi Arabia to adopt a system-wide approach and focus its local resources spending on more efficient pharmacological and non-pharmacological interventions, to reduce the population obesity burden.

Furthermore, few studies in our review have examined differences in obesity among provinces and multiple age groups in Saudi Arabia. Although several studies have examined the association of obesity with various aspects of ORCs, the number of studies that reported the prevalence of ORCs in PwO was limited. These data were reported only for T2D, CVD, sleep disorders, infertility, PCOS, osteoarthritis, and poor mental health in the studies identified. Other ORCs were not studied or reported in Saudi Arabia. Therefore, future research should target these ORCs in Saudi Arabia to provide insights and complement the current understanding of obesity burden in the country.

The lack of advanced technologies for obesity care and management in the Saudi Arabian healthcare system is partly attributable to the current obesity pandemic (AlZughbi et al., 2018). The adoption of advanced electronic medical records and artificial intelligence (Chew et al., 2021), as well as training to implement comparable assessments of obesity and its complications across the healthcare system, would enable the collation of real-world data, which would greatly aid in the understanding and management of obesity and its complications in Saudi Arabia (Alqahtani et al., 2023). Further research on the impact of technological advancements, such as integrating artificial intelligence techniques with machine-learning algorithms in electronic medical records to screen and predict people at high risk of developing obesity and OCRs, based on patients’ clinical characteristics.

A limitation of this study was the restriction of including research published between 2012 and 2022. Although this ensured that the studies listed in the SLR were the most recent, there may have been other relevant studies published before 2012 or after 2022 that needed to be listed in the SLR. In addition, different methodologies, study designs, and data sources varied across studies, which may have an impact on the findings consistency for included studies and may have limited the generalizability of the SLR results. However, the SLR did provide a comprehensive overview of the prevalence of obesity among adults and children, males and females in Saudi Arabia and demonstrated the clinical and economic implications of obesity both in the community and in the healthcare system. Future research focusing on action plans and tactics is needed in order to close the gap of evidence and to support the mutual efforts of several stakeholders in fighting obesity.

## 5 Conclusion

Obesity and its related complications can significantly impact PwO both clinically and economically in addition to having a detrimental impact on society, healthcare system services, and allocated resources. Therefore, it is crucial to understand the prevalence of obesity and its impact on health and morbidity, the healthcare system, healthcare financial resources, resource utilization, and the economic burden, in order to develop and implement effective management strategies for obesity. This review provides a holistic view of the prevalence of obesity and its associated complications in Saudi Arabia. In addition, it identifies key evidence gaps regarding healthcare resource utilization and the economic burden of obesity in Saudi Arabia. Although the outcomes from this SLR are promising, additional research is required to increase understanding of this disease. Further research also needs to focus on developing national strategies and interventions for obesity and ORCs that operate at multiple levels and target both individuals and communities. The results from such research would benefit individuals, society, and policymakers, and be essential in transforming and supporting the healthcare system and policy in alignment with Saudi Arabia’s Vision 2030 objectives.

## Disclosure

Ali Alshehri has provided consulting services to Novo Nordisk. Mahmoud S. Eldin is employee of Novo Nordisk in Saudi Arabia. Hussain A. Al-Omar, Hana Alabdulkarim, Ali Alrumaih, and Saleh A. Alqahtani have nothing to disclose.

## CRediT authorship contribution statement

**Hussain A. Al-Omar:** Writing – review & editing, Writing – original draft, Supervision, Methodology, Formal analysis, Data curation, Conceptualization. **Ali Alshehri:** Writing – review & editing, Writing – original draft, Methodology, Data curation, Conceptualization. **Saleh A. Alqahtani:** Writing – review & editing, Writing – original draft, Supervision, Methodology, Conceptualization. **Hana Alabdulkarim:** Writing – review & editing, Conceptualization. **Ali Alrumaih:** Writing – review & editing, Writing – original draft, Methodology, Data curation, Conceptualization. **Mahmoud S. Eldin:** Writing – review & editing, Writing – original draft, Formal analysis, Data curation, Conceptualization.

## Funding

This study was funded by Novo Nordisk, Saudi Arabia.
