# Supplementary material for: A systematic review of obesity burden in Saudi Arabia: Prevalence and associated co-morbidities
Source: Saudi Pharm J. 2024 Oct 24;32(11):102192. doi: 10.1016/j.jsps.2024.102192 (PMC11550078; doi:10.1016/j.jsps.2024.102192)
Supplement: Supplementary Data 1 [file mmc1.docx]

# Supplementary Material

## Search strategy

PubMed, EMBASE, and IFSO 2018 congress abstract searches

PICOS criteria

| Population | Children and adults in Saudi Arabia |
| --- | --- |
| Intervention | Any |
| Comparator | Any |
| Outcomes | Prevalence of obesity  Prevalence of obesity-related complications |
| Study | Any study design |

PICOS, population, intervention, comparator, outcomes, and study
