# Supplementary material for: A systematic review of obesity burden in Saudi Arabia: Prevalence and associated co-morbidities
Source: Saudi Pharm J. 2024 Oct 24;32(11):102192. doi: 10.1016/j.jsps.2024.102192 (PMC11550078; doi:10.1016/j.jsps.2024.102192)
Supplement: Supplementary Data 2 [file mmc2.docx]

**Appendix A: Search strategy and used keywords in EMBASE and PubMed.**

| **Search strategy for EMBASE** | | |
| --- | --- | --- |
| No. | Query | Results |
| #1 | 'obesity'/exp/mj OR 'obesity':ti OR 'obese':ti | 284286 |
| #2 | saudi arabia'/exp OR 'saudi arabia':ti,ab OR 'kingdom of saudi arabia':ti,ab OR 'ksa':ti,ab OR 'saudi':ti,ab OR 'saudis':ti,ab OR 'arab':ti,ab OR 'arabs':ti,ab OR 'arabia':ti,ab OR 'arabian':ti,ab OR 'arabic':ti,ab OR 'gcc':ti,ab OR 'gulf cooperation council':ti,ab OR 'kuwait'/exp OR 'kuwait':ti,ab OR 'kuwaiti':ti,ab OR 'kuwaitis':ti,ab OR 'bahrain'/exp OR 'bahrain':ti,ab OR 'bahraini':ti,ab OR 'bahrainis':ti,ab OR 'qatar'/exp OR 'qatar':ti,ab OR 'qatari':ti,ab OR 'qataris':ti,ab OR 'united arab emirates'/exp OR 'united arab emirates':ti,ab OR 'uae':ti,ab OR 'emirati':ti,ab OR 'emiratis':ti,ab OR 'oman'/exp OR 'oman':ti,ab OR 'omani':ti,ab OR 'omanis':ti,ab OR 'yemen':ti,ab OR 'yemenis':ti,ab OR 'yemeni':ti,ab OR 'yemen'/exp OR 'gulf':ti,ab | 89229 |
| #3 | #1 AND #2 | 1539 |
| #4 | epidemiology'/exp OR 'epidemiology' OR 'epidemiologic' OR 'incidence'/exp OR 'incidence' OR 'prevalence'/exp OR 'prevalence' OR 'rate' OR 'rates' OR 'proportion' OR 'proportions' | 8627992 |
| #5 | economics'/exp OR 'economic' OR 'economics' OR 'economically' OR 'cost' OR 'costs' OR 'price' OR 'prices' OR 'pricing' OR 'expenditure' OR 'expenditures' OR 'fee' OR 'fees' OR 'charge' OR 'charges' OR 'debt' OR 'debts' OR 'fund' OR 'funds' OR 'funded' OR 'funding' OR 'pay' OR 'payer' OR 'payers' OR 'insurance' OR 'reimbursement' OR 'income' OR 'salary' OR 'revenue' OR 'finance' OR 'finances' OR 'financing' OR 'financial' OR 'financially' OR 'gross domestic product' OR 'gdp' OR 'spend' OR 'spends' OR 'spending' OR 'expense' OR 'resource use utilisation' OR 'resource use utilization' OR 'ruu' OR 'expenses' | 2539904 |
| #6 | 'morbidity'/exp OR 'morbidity' OR 'comorbidity'/exp OR 'comorbidity' OR 'comorbidities' OR 'comorbid' OR 'co-morbidity' OR 'co-morbidities' OR 'co-morbid' OR 'multimorbidity' OR 'multi-morbidity' OR 'complication' OR 'complications' OR 'risk factor'/exp OR 'risk factor' OR 'risk factors' OR 'health correlate' OR 'health correlates' OR 'health consequence' OR 'health consequences' | 5055952 |
| #7 | 'cardiometabolic' OR 'cardio metabolic' OR 'cardiovascular disease'/exp OR 'metabolic disorder'/exp OR 'nafld' OR 'non-alcoholic fatty liver disease' OR 'nonalcoholic fatty liver'/exp OR 'non alcoholic fatty liver disease' OR 'nonalcoholic fatty liver disease' OR 'sleep apnoea' OR 'sleep apnea' OR 'sleep disordered breathing'/exp OR 'osteoarthritis' OR 'osteoarthritis'/exp OR 'stroke' OR 'cerebrovascular accident'/exp | 6897950 |
| #8 | #4 OR #5 OR #6 OR #7 | 16219537 |
| #9 | #3 AND #8 | 1413 |
| #10 | #3 AND #8 AND [2010-2021]/py | 1088 |
| #11 | #4 AND #10 | 889 |
| #12 | #5 AND #10 | 162 |
| #13 | #6 AND #10 | 515 |
| #14 | #7 AND #10 | 689 |
| **Search strategy for Pubmed** | | |
| No. | Query | Results |
| #1 | Search: "Obesity"[MAJR] OR "Obesity"[ti] OR "Obese"[ti] Sort by: Publication Date | 183,453 |
| #2 | Search: "Saudi Arabia"[Mesh] OR "Saudi Arabia"[tiab] OR "Kingdom of Saudi Arabia"[tiab] OR "KSA"[tiab] OR "Saudi"[tiab] OR "Saudis"[tiab] OR "Arab"[tiab] OR "Arabs"[tiab] OR "Arabia"[tiab] OR "Arabian"[tiab] OR "Arabic"[tiab] OR "GCC"[tiab] OR "Gulf Cooperation Council"[tiab] OR "Kuwait"[Mesh] OR "Kuwait"[tiab] OR "Kuwaiti"[tiab] OR "Kuwaitis"[tiab] OR "Bahrain"[Mesh] OR "Bahrain"[tiab] OR "Bahraini"[tiab] OR "Bahrainis"[tiab] OR "Qatar"[Mesh] OR "Qatar"[tiab] OR "Qatari"[tiab] OR "Qataris"[tiab] OR "United Arab Emirates"[Mesh] OR "United Arab Emirates"[tiab] OR "UAE"[tiab] OR "Emirati"[tiab] OR "Emiratis"[tiab] OR "Oman"[Mesh] OR "Oman"[tiab] OR "Omani"[tiab] OR "Omanis"[tiab] OR "Yemen"[tiab] OR "Yemenis"[tiab] OR "Yemeni"[tiab] OR "Yemen"[Mesh] OR "Gulf"[tiab] Sort by: Publication Date | 73,343 |
| #3 | Search: #1 AND #2 Sort by: Publication Date | 953 |
| #4 | Search: "Epidemiology"[MeSH] OR "Epidemiology"[subheading] OR "Epidemiology"[all] OR "Epidemiologic"[all] OR "Incidence"[all] OR "Prevalence"[all] OR "Rate"[all] OR "Rates"[all] OR "Incidence"[MeSH] OR "Prevalence"[MeSH] OR "Proportion"[all] OR "Proportions"[all]  Sort by: Publication Date | 5,934,965 |
| #5 | Search: "Economics"[MeSH] OR "Economic"[all] OR "Economics"[all] OR "Economically"[all] OR "Cost"[all] OR "Costs"[all] OR "Price"[all] OR "Prices"[all] OR "Pricing"[all] OR "Expenditure"[all] OR "Expenditures"[all] OR "Fee"[all] OR "Fees"[all] OR "Charge"[all] OR "Charges"[all] OR "Debt"[all] OR "Debts"[all] OR "Fund"[all] OR "Funds"[all] OR "Funded"[all] OR "Funding"[all] OR "Pay"[all] OR "Payer"[all] OR "Payers"[all] OR "Insurance"[all] OR "Reimbursement"[all] OR "Income"[all] OR "Salary"[all] OR "Revenue"[all] OR "Finance"[all] OR "Finances"[all] OR "Financing"[all] OR "Financial"[all] OR "Financially"[all] OR "Gross Domestic Product"[all] OR "GDP"[all] OR "Spend"[all] OR "Spends"[all] OR "Spending"[all] OR "Expense"[all] OR "Resource Use Utilisation"[all] OR "Resource Use Utilization"[all] OR "RUU"[all] OR "Expenses"[all] Sort by: Publication Date | 2,269,848 |
| #6 | Search: "Morbidity"[MeSH] OR "Morbidity"[all] OR "Comorbidity"[MeSH] OR "Comorbidity"[all] OR "Comorbidities"[all] OR "Comorbid"[all] OR "Co-morbidity"[all] OR "Co-morbidities"[all] OR "Co-morbid"[all] OR "Multimorbidity"[all] OR "Multi-morbidity"[all] OR "Complication"[all] OR "Complications"[all] OR "Health correlate"[all] OR "Health correlates"[all] OR "Risk factors"[MeSH] OR "Risk factor"[all] OR "Risk factors"[all] OR "Health consequence"[all] OR "Health consequences"[all] Sort by: Publication Date | 4,598,446 |
| #7 | Search: "Cardiometabolic"[all] OR "cardio metabolic"[all] OR "Cardiovascular diseases"[MeSH] OR "Metabolic diseases"[MeSH] OR "NAFLD"[all] OR "Non-alcoholic fatty liver disease"[all] OR "Non-alcoholic Fatty Liver Disease"[MeSH] OR "Non alcoholic fatty liver disease"[all] OR "Nonalcoholic fatty liver disease"[all] OR "Sleep apnoea"[all] OR "Sleep apnea"[all] OR "Sleep Apnea Syndromes"[MeSH] OR "Osteoarthritis"[all] OR "Osteoarthritis"[Mesh] OR "Stroke"[all] OR "Stroke"[Mesh] Sort by: Publication Date | 3,549,646 |
| #8 | Search: #3 AND (#4 OR #5 OR #6 OR #7) | 838 |
| #9 | Search: #3 AND (#4 OR #5 OR #6 OR #7) | 627 |
| #10 | Search: #4 AND #9 | 512 |
| #11 | Search: #5 AND #9 | 118 |
| #12 | Search: #6 AND #9 | 430 |
| #13 | Search: #7 AND #9 | 243 |
