# Supplementary material for: A systematic review of obesity burden in Saudi Arabia: Prevalence and associated co-morbidities
Source: Saudi Pharm J. 2024 Oct 24;32(11):102192. doi: 10.1016/j.jsps.2024.102192 (PMC11550078; doi:10.1016/j.jsps.2024.102192)
Supplement: Supplementary Data 3 [file mmc3.docx]

**Appendix B: Overall risk assessment table for included studies.**

| **Study** | **Type of study design** | **Overall Risk of**  **Bias Assessment** | **Study** | **Type of study design** | **Overall Risk of Bias Assessment** |
| --- | --- | --- | --- | --- | --- |
| Elkum et al., 2014 | Case-control | Low | Alshehri et al., 2019 | Cross-sectional | Low |
| Ibrahim et al., 2017 | Case-control | Low | Alsibyani et al., 2017 | Cross-sectional | Low |
| Abohamr et al., 2020 | Case-series | Low | Al-Sumaih et al., 2020 | Cross-sectional | Low |
| Al-Nakeeb et al., 2012 | Cross-sectional | Low | Althumiri et al., 2021 | Cross-sectional | Low |
| Ahmed et al., 2017 | Cross-sectional | Low | Alwan İbrahim et al., 2013 | Cross-sectional | Low |
| Al-Agha et al., 2016a | Cross-sectional | Low | Alwasaidi et al., 2017 | Cross-sectional | Low |
| Al-Agha et al., 2016b | Cross-sectional | Low | Al-Zahrani et al., 2019 | Cross-sectional | Low |
| Alammar et al., 2020 | Cross-sectional | Low | Alzeidan et al., 2020 | Cross-sectional | Low |
| Alanazi et al., 2022 | Cross-sectional | Low | Ashi et al., 2019 | Cross-sectional | Low |
| AlBuhairan et al., 2015 | Cross-sectional | Low | Assakran et al., 2020 | Cross-sectional | Low |
| Al-Daghri et al., 2015 | Cross-sectional | Low | Azzeh et al., 2017 | Cross-sectional | Low |
| Aldiab et al., 2018 | Cross-sectional | Low | Bandy et al., 2019 | Cross-sectional | Low |
| Al-Enazy et al., 2014 | Cross-sectional | Low | Elkhodary and Farsi, 2017 | Cross-sectional | Low |
| Alghamdi et al., 2017 | Cross-sectional | Low | Faisal et al., 2016 | Cross-sectional | Low |
| Alghnam et al., 2021 | Cross-sectional | Low | Farsi and Elkhodary, 2017 | Cross-sectional | Low |
| Alhazmi et al., 2017 | Cross-sectional | Low | Farsi et al., 2016 | Cross-sectional | Low |
| Al-Hazzaa et al., 2014 | Cross-sectional | Low | Habib, 2013 | Cross-sectional | Low |
| Al-Hussaini et al., 2019 | Cross-sectional | Low | Memish et al., 2014 | Cross-sectional | Low |
| Al-Hussein et al., 2014 | Cross-sectional | Low | Mosli et al., 2020 | Cross-sectional | Low |
| Aljabri et al., 2018 | Cross-sectional | Low | Nasim et al., 2019 | Cross-sectional | Low |
| Al-Kadi et al., 2018 | Cross-sectional | Low | Shaikh et al., 2016 | Cross-sectional | Low |
| Al-Kutbe et al., 2017 | Cross-sectional | Low | Wagdy et al., 2021 | Cross-sectional | Low |
| AlKuwaity et al., 2018 | Cross-sectional | Low | Aleidi et al., 2021 | Experimental | Low |
| Almaawi et al., 2020 | Cross-sectional | Low | Alqahtani et al., 2022 | Prospective | Low |
| Almarhoon et al., 2021 | Cross-sectional | Low | Alshahrani et al., 2016 | Prospective | Low |
| Al-Mohaimeed et al., 2015 | Cross-sectional | Low | Altamimi et al., 2020 | Prospective | Low |
| Al-Mohaimeed, 2016 | Cross-sectional | Low | Mobeirek et al., 2014 | Prospective | Low |
| Almojarthe et al., 2020 | Cross-sectional | Low | Alguwaihes et al., 2020 | Retrospective | Low |
| Al-Nakeeb et al., 2012 | Cross-sectional | Low | Alharbi et al., 2022 | Retrospective | Low |
| Al-Omari et al., 2020 | Cross-sectional | Low | AlJabr et al., 2021 | Retrospective | Low |
| AlQahtani et al., 2015 | Cross-sectional | Low | AlKhafaji et al., 2022 | Retrospective | Low |
| Al-Qahtani, 2019 | Cross-sectional | Low | Alqahtani et al., 2012 | Retrospective | Low |
| Al-Raddadi et al., 2019 | Cross-sectional | Low | Alqahtani et al., 2022 | Retrospective | Low |
| Alsabaani et al., 2018 | Cross-sectional | Low | Al-Ruthia et al., 2017 | Retrospective | Low |
| Alsaghah et al., 2019 | Cross-sectional | Low | Al-Saleh et al., 2019 | Retrospective | Low |
| Alsaleem, 2021 | Cross-sectional | Low | Alshamsan et al., 2022 | Retrospective | Low |
| AlShahrani, 2021 | Cross-sectional | Low | Melebari et al., 2021 | Retrospective | Low |
| Alshammari et al., 2017 | Cross-sectional | Low | Rafique and Nuzhat, 2016 | Retrospective | Low |
| Rasmy and Sorour, 2020 | Retrospective | Low | Rafique et al., 2021 | Retrospective | Low |
| Ahmed et al., 2014 | Survey | Low |  |  |  |
